# Grand Challenges in Musculoskeletal Pain Research: Chronicity, Comorbidity, Immune Regulation, Sex Differences, Diagnosis, and Treatment Opportunities

**DOI:** 10.3389/fpain.2020.575479

**Published:** 2020-10-23

**Authors:** Ke Ren

**Affiliations:** ^1^Department of Neural and Pain Sciences, School of Dentistry, University of Maryland, Baltimore, MD, United States; ^2^Program in Neuroscience, University of Maryland, Baltimore, MD, United States

**Keywords:** chronic pain, muscle pain, joint pain, widespread pain, comorbid pain, stem cell therapy

## Introduction

Musculoskeletal pain involves bones, joints, and related muscular tissues and includes most debilitating pain conditions such as low back pain, arthritic pain, and widespread muscle pain. Chronic musculoskeletal pain is the most predominant among chronic pain conditions and presents a serious challenge to primary care. Pain arising from musculoskeletal tissues is characteristic of deep pain and has important differences from that of cutaneous pain [Table 1; [1]]. Compared to cutaneous tissue injury, the injection of the same amount of inflammatory agent into the joint induces more intense inflammation and greater and more widespread neuronal activation in the pain transmission pathways [2]. Musculoskeletal nociceptive inputs appear more effective in inducing neuronal excitation and produce greater sensory disturbances [3, 4], which may explain predominant chronic pain conditions involving deep tissues. The underlying etiology and pathology of chronic musculoskeletal pain conditions are poorly understood even after decades of research.

**Table 1:** Comparison of musculoskeletal and cutaneous pain.

	**Musculoskeletal pain**	**Cutaneous pain**
Localization	Diffuse	Localized
Description	Cramping, aching, throbbing, dull	Sharp, pricking, stabbing, shooting
Noxious St.	Not necessarily tissue-damaging	Usually tissue-damaging
Referred pain	Yes	No
Hyperalgesia/Allodynia	Yes	Yes

## Experimental Musculoskeletal Pain - Relevance to Chronicity

Human muscle pain can be induced experimentally by injecting the hyperalgesic agents such as hypertonic saline [5], capsaicin [6], glutamate [7], serotonin [8], or nerve growth factor [9, 10] into the muscle. To avoid invasive procedures, a short-wave diathermy-induced human muscle pain model has been developed [11]. These human muscle pain models help to advance our understanding of the mechanisms and improve the treatment of muscle pain. A limitation of these models, however, is that the induced muscle pain hypersensitivity would resolve within minutes to hours [e.g., [5, 12]]. Nerve growth factor induces relatively long-lasting hyperalgesia for 4–7 days to a few weeks after injection into the muscle in humans [9, 13, 14]. Chronic pain is defined as pains that persist or recur for longer than 3 months [15]. While acute and chronic pain shares similar neural mechanisms, chronic pain is underlying by distinct mechanisms of central sensitization and still unsettled involvement of the immune system [16–18]. Although these human experimental models mimic aspects of acute or persistent pain, they are not necessarily suitable for studying pain chronicity, a deteriorating and devastating problem for patients.

Animal persistent musculoskeletal pain models have been developed to simulate chronic pain conditions in humans. A variety of methods can be used to assess muscle/joint pain in animals, including evoked nocifensive reflex and vocalization, movement-related measures such as weight-bearing and gait analysis, functional measures such as bite force and grip force, spontaneous nociception such as home cage monitoring, scratching behaviors, and grimace scale. To mimic human rheumatic disease, polyarthritis was induced by injecting complete Freund's adjuvant (CFA) into the base of the rat's tail [19]. Pain hypersensitivity occurs in multiple joints after 10 days and lasts up to 3 weeks. Later studies revealed that a systemic disease was induced in this model that included skin lesions, destruction of bone and cartilage, impairment of liver function, and lymphadenopathy, which made it difficult to differentiate pain behavior from generalized malaise and debilitation, and led to ethical concerns [20]. Injection of inflammatory irritants into the joints and muscles, including CFA, carrageenan, zymosan, mustard oil, formalin, capsaicin, bee venom, acidic saline, lipopolysaccharide, inflammatory cytokines, monosodium iodoacetate, and sodium urate crystals, has been used to produce tissue injury and hyperalgesia [[21], review]. These models mainly reflect early injury responses and there is still a significant challenge to develop models that can be translated into human *chronic* musculoskeletal pain conditions [see [22]].

Surgical interventions are employed to model chronic low back pain, the most commonly seen chronic musculoskeletal pain [reviewed in [23]]. The procedures include the compression of the dorsal root ganglion [24], disruption of the lumbar intervertebral disc [25], implantation of tissues into the lumbar epidural space to mimic disc herniation [26], injection of complete Freund's adjuvant into the intervertebral disc or nucleus pulposus [27], and surgical application of zymosan into the epidural space to induce inflammation of the dorsal root ganglion [28]. These approaches lead to behavioral hyperalgesia resembling human low back pains such as discogenic and radicular back pain and low back pain related to local inflammation. The pain hypersensitivity after surgical interventions can last for up to months [24, 25, 27], longer than that after a simple injection of algesic mediators. To mimic soft tissue musculoskeletal pain such as tendinopathy, a constriction injury of the tendon of the rat masseter muscle produces pain hypersensitivity that lasts for months [29, 30]. These models may be employed to identify distinct mechanisms that contribute to the development of *chronic* musculoskeletal pain.

The new International Classification of Diseases-11 has updated the chronic pain classification to include the *Primary* chronic pain category to designate chronic pain that cannot directly be ascribed to any disease of structural injury. Fibromyalgia, a chronic condition characterized by widespread pain involving musculoskeletal tissues, is a type of primary chronic musculoskeletal pain and women appear to be affected more than men [31, 32]. A reserpine myalgia model has been presented to model fibromyalgia in animals [33]. In this model, a daily subcutaneous injection of reserpine (1 mg/kg) was repeated for 3 consecutive days induced decreased muscle pressure threshold and allodynia. While the reserpine approach is promising, the model's relevance to fibromyalgia is still an issue. The reserpine-induced myalgia would only persist for about 1 week and the sex difference in fibromyalgia prevalence is not reproduced [33, 34]. Reserpine depletes monoamine neurotransmitters norepinephrine, dopamine, and serotonin by inhibiting vesicular monoamine transporters [35]. Reserpine-induced pain suggests that these monoamines exert a net inhibitory effect on nociception. It is interesting to note that Catechol-O-methyltransferase (COMT) metabolizes catecholamines, but inhibition of COMT leads to enhanced catecholamine levels and pain hypersensitivity via beta2/3-adrenoceptors [36]. The imbalance of monoamines in fibromyalgia and functional pain syndrome requires further investigation.

The preclinical muscle/bone/joint pain models have contributed greatly to our understanding of the biological mechanisms underlying musculoskeletal pain. However, there are still gaps in our understanding, particularly with the development of pain chronicity. Most studies have settled to use the observed *persistent* pain within days or a couple of weeks after the injury as a surrogate of chronic pain in humans, despite that the early persistent pain could still be a type of acute pain and may miss characteristics of chronic pain that occurs late. Recent studies have reported month-long upregulation of microglial markers in the spinal cord after nerve injury and differential cytokine profiles between the early and late phases of hyperalgesia [37]. The transcription factor NF-kB is known for its immediate pro-inflammatory role, but it also contributes to the resolution of inflammation at the *late* phase of inflammation [38]. Reevaluation of the CFA inflammatory hyperalgesia model indicates that the temporal course of mechanical hyperalgesia consists of an initial *developing* phase with peak hyperalgesia at 4–24 h, a subsequent *attenuating* phase of a few weeks, followed by a late *persistent* (chronic) phase that lasted for months [18]. Importantly, different cellular mechanisms are involved in the early acute phase and late chronic phase of CFA-induced hyperalgesia, as suggested by a late downregulation of astroglial glutamate transporters that occurs at a time when hyperalgesia transitions into the persistent chronic phase [18]. Thus, preclinical studies on chronic musculoskeletal pain need to attend to the late chronic phase of pain hypersensitivity. The central mechanisms involved in the transition of chronic muscle pain should also be studied [39, 40]. We are challenged to differentiate the factors relevant to the transition and maintenance of musculoskeletal pain chronicity. Myalgia is one of the major symptoms of Covid-19 [41, 42]. It remains to be seen whether it could develop into a chronic problem.

## Widespread and Comorbid Pain Conditions

Widespread pain is commonly seen in musculoskeletal pain conditions, which is characteristic of deep tissue pain that refers to the areas remote from injury. The temporomandibular joint disorders (TMJD) patients not only have pain in the TMJ and muscles of mastication but also pain in other muscles and joints [43, 44]. Somewhat conceptually overlapping with widespread pain, patients with chronic musculoskeletal pain frequently have other comorbid pain conditions: fibromyalgia and TMJD [45, 46], migraine [47], and visceral pain [48], TMJD and headache [49], and Ehlers-Danlos syndromes (joint pain) and Chiari Malformation (headache and neck pain) [50]. A causal relationship between the comorbid pain conditions is often difficult to determine regarding which condition triggers the other, but the involvement of multiple body structures suggests a central-mediated effect [51, 52].

Widespread and comorbid muscle pains can be reproduced in animal models. Repeated unilateral injections of acidic saline into the gastrocnemius muscle of rats produce bilateral hyperalgesia that lasts for up to 30 days [53], which mimics persistent widespread muscle pain in humans. Unilateral injection of CFA into the masseter muscle induces bilateral behavioral hyperalgesia [54]. Combined masseter muscle inflammation and stress induce visceral hypersensitivity similar to that seen in comorbid TMJD and irritable bowel syndrome patients [55]. Interestingly, the inflammatory pain of the craniofacial muscle in rats can spread to the hind paw, but not vice versa [56]. Since primary sensory afferents from the craniofacial region project to a wide region of the brain, in contrast to distinct somatotopy of spinal afferents [57], craniofacial musculoskeletal pain tends to induce comorbid pain conditions. The cellular mechanisms underlying the comorbidity of chronic musculoskeletal pain remain to be elucidated.

## Immune Regulation in Persistent Pain

The development of persistent or chronic pain largely depends on the interactions between the nervous and immune systems, which involves glia that function as immune cells in the brain [17, 58]. The pain-related neuroimmune interactions are reciprocal and involve neurotransmitters and their receptors and immune mediators including cytokines and their receptors. In inflammatory hyperalgesia after injection of CFA into the masseter muscle, there is reactive astrogliosis, induction of proinflammatory cytokine IL-1b, and the coupling of NMDA receptor phosphorylation through IL-1 receptor signaling [59]. In ischemic myalgia, IL-1b signals through IL-1 receptor to upregulate acid-sensing ion channels to induce nociceptor sensitization [60]. Activation of P2X4 receptors on muscle macrophages leads to IL-1b release and muscle hyperalgesia [61].

Despite evidence from preclinical studies, clinical trials for the treatment of chronic pain with glial modulators have been unsuccessful [62], which would suggest our incomplete understanding of the mechanisms. From imaging studies on chronic low back pain patients, it is observed that glial activity is negatively correlated with the levels of pain and IL-1b, suggesting an inhibitory role of glia related to the translocator protein, the marker used for glial activation in the study [63]. This inhibitory role of glial activity has largely been overlooked. The immune system provides balanced regulation to maintain normal function. The effector and regulatory T cells (Teffs and Tregs), for example, are pro- and anti-inflammatory, respectively [64]. The depletion of Tregs delays pain resolution [65] and enhances neuropathic pain [66]. Tumor necrosis factor (TNF) is proinflammatory through the TNFR1 while anti-inflammatory via TNFR2 [67]. Deletion of TNFR2 hampers, but activation of TNFR2 promotes recovery from neuropathic pain [65]. The known proinflammatory NF-kB pathway is involved in the development of pain hypersensitivity but also involved in bone marrow stromal cell-produced pain relief in a tendinopathy model [68], suggesting dual roles of NF-kB in hyperalgesia and pain relief according to circumstances. These results suggest that the perturbation of the balanced or homeostatic immune regulation, not only immune activation, leads to disease conditions including chronic pain. In search of treatment strategies targeting the immune system, it would be ideal to return to homeostasis by strengthening the anti-inflammatory/protective profile, instead of simply shutting off immune activation.

## Sex Differences

The phenomenon of female predominance in chronic pain has been a topic of interest in recent decades [69–71], which has led to the National Institutes of Health (NIH) mandate to include sex as a biological variable in NIH-funded Research [72]. Similar to other chronic pain conditions, women appear to be affected more than men by chronic and co-morbid musculoskeletal pain [73, 74]. While the relative prevalence of chronic pains in females and males can be quantified, we still need to find answers for the factors that contribute to these differences. In addition to document different levels of pain, more focus should be on the underlying differences in biology between males and females. Preclinical studies have shown male-specific involvement of Toll-like receptor 4 in persistent pain [see [71]]. A recent report shows that the upregulation of genes that escape X chromosome inactivation is correlated to the development of co-morbid chronic musculoskeletal pain after a car accident in women, but not in men [74]. Elucidating the underlying differential biology in sex differences in pain would be important in avoiding the biased development of pain medicine.

## Diagnosis and Etiology

Most of our knowledge about mechanisms of persistent pain is based on studies of cutaneous pain in animals, experimental subjects, and patients. Although it is tempting to generalize these findings to the pain of deep tissues such as muscle and joint, there are important differences between cutaneous and deep pain (Table 1). Deep pains are diffuse and difficult to localize and represent a challenge in diagnosis and identification of etiology. It is often difficult to identify the direct cause of chronic musculoskeletal pain conditions such as TMJD and low back pain [75, 76]. The diagnosis and etiology of primary chronic musculoskeletal pain, fibromyalgia in particular [77], are still under debate. Clinical studies in this area are much needed to improve the prevention and treatment of chronic musculoskeletal pain.

## Treatment Opportunities

Managing musculoskeletal pain, especially when it becomes chronic, is a daily challenge in primary care. Commonly used pharmacological agents are NSAIDs, opioids, and steroids. Treatment options also include physical therapy, psychotherapy, mesotherapy, whole-body cryotherapy, and alternative or complementary therapies acupuncture, prolotherapy, percutaneous electrical nerve stimulation, and neuromuscular electrical stimulation. The current treatment provides some relief of acute or short-lasting pain but is unsatisfactory for chronic musculoskeletal pain [78–81]. A recent report showed that transcranial magnetic stimulation of the prefrontal cortex attenuated long-term experimental muscle pain in human subjects [14].

Cell-based therapy has shown tremendous promise in the management of chronic musculoskeletal pain in recent years. Patients with disc diseases receiving intradiscal injection of bone marrow concentrate show improvement of discogenic pain through up to a 3-year follow-up [82, 83]. The clinical improvement is attributable to mesenchymal stromal cells (MSCs) in the bone marrow. Clinical studies have shown the pain-reducing effect of multipotent MSCs in arthritic joint pain [84–86], rotator cuff disease [87], and discogenic pain [88, 89]. Large scale randomized controlled trials are necessary to substantiate these exciting findings.

Preclinical studies have addressed cellular mechanisms of MSC-produced musculoskeletal pain relief. In a tendon injury model, a single intravenous injection of bone marrow-derived MSCs produces long-term attenuation of behavioral hyperalgesia [30, 90]. The pain-attenuation is induced through the interaction of BMSCs with immune cells and mediators, that lead to suppression of proinflammatory cytokines and upregulation of anti-inflammatory cytokines [91], inhibition of NMDA receptor phosphorylation [92], and activation of endogenous opioid receptors [93]. One pitfall of systemic MSCs is that they tend to be trapped mostly in the lungs after transplantation [94, 95] and there is a case of reversible pulmonary embolism after multiple infusion of adipose tissue-derived MSCs [96]. However, MSCs injected locally to the spinal cord or transplanted directly into degenerated disc appeared to survive longer [97, 98]. The therapeutic effect of MSCs can be further improved by modifying their phenotype before transplantation [99, 100]. Studies on the use of therapeutic MSCs are rapidly expanding and we are looking forward to the establishment of novel treatment strategies for chronic musculoskeletal pain.

## Author Contributions

KR confirms being the sole contributor to this work and approved it for publication.

## Conflict of Interest

The author declares that the research was conducted in the absence of any commercial or financial relationships that could be construed as a potential conflict of interest.

## References

[1] Dubner R. Basic mechanisms of pain associated with deep tissues. Can J Physiol Pharmacol. (1991) 69:607–9. 10.1139/y91-090 1863910

[2] Imbe H Iwata K Zhou Q. -Q. Zou S Dubner R. Orofacial deep and cutaneous tissue inflammation and trigeminal neuronal activation: Implications for persistent temporomandibular pain. Cells Tissues Organs. (2001) 169:238–47. 10.1159/000047887 11455119

[3] Wall PD Woolf CJ. Muscle but not cutaneous C-afferent input produces prolonged increases in the excitability of the flexion reflex in the rat. J Physiol. (1984) 356:443–58. 10.1113/jphysiol.1984.sp015475 6520794 PMC1193174

[4] Yu X-M Sessle BJ Hu JW. Differential effects of cutaneous and deep application of inflammatory irritant on mechanoreceptive field properties of trigeminal brain stem nociceptive neurons. J Neurophysiol. (1993) 70:1704–7. 10.1152/jn.1993.70.4.1704 8283224

[5] Veerasarn P Stohler CS. The effect of experimental muscle pain on the background electrical brain activity. Pain. (1992) 49:349–60. 10.1016/0304-3959(92)90242-4 1408301

[6] Arima T Svensson P Arendt-Nielsen L. Capsaicin-induced muscle hyperalgesia in the exercised and non-exercised human masseter muscle. J Orofac Pain. (2000) 14:213–23. 11203756

[7] Castrillon EE Cairns BE Ernberg M Wang K Sessle B Arendt-Nielsen L. Glutamate-evoked jaw muscle pain as a model of persistent myofascial TMD pain? Arch Oral Biol. (2008) 53:666–76. 10.1016/j.archoralbio.2008.01.008 18313028 PMC2491493

[8] Ernberg M Lundeberg T Kopp S. Pain and allodynia/hyperalgesia induced by intramuscular injection of serotonin in patients with fibromyalgia and healthy individuals. Pain. (2000) 85:31–9. 10.1016/S0304-3959(99)00233-X 10692600

[9] Svensson P Cairns BE Wang K Arendt-Nielsen L. Injection of nerve growth factor into human masseter muscle evokes long-lasting mechanical allodynia and hyperalgesia. Pain. (2003) 104:241–7. 10.1016/S0304-3959(03)00012-5 12855334

[10] Svensson P Castrillon E Cairns BE. Nerve growth factor-evoked masseter muscle sensitization and perturbation of jaw motor function in healthy women. J Orofac Pain. (2008) 22:340–8. 19090407

[11] Mista CA Laugero SJ Adur JF Andersen OK Biurrun Manresa JA. A new experimental model of muscle pain in humans based on short-wave diathermy. Eur J Pain. (2019) 23:1733–42. 10.1002/ejp.1449 31251430

[12] Ro JY Lee JS Zhang Y. Activation of TRPV1 and TRPA1 leads to muscle nociception and mechanical hyperalgesia. Pain. (2009) 144:270–7. 10.1016/j.pain.2009.04.021 19464796 PMC2789550

[13] Bergin MJ Hirata R Mista C Christensen SW Tucker K Vicenzino B. Movement evoked pain and mechanical hyperalgesia after intramuscular injection of nerve growth factor: a model of sustained elbow pain. Pain Med. (2015) 16:2180–91. 10.1111/pme.12824 26178748

[14] Seminowicz DA de Martino E Schabrun SM Graven-Nielsen T. Left dorsolateral prefrontal cortex repetitive transcranial magnetic stimulation reduces the development of long-term muscle pain. Pain. (2018) 159:2486–92. 10.1097/j.pain.0000000000001350 30431520

[15] Treede RD Rief W Barke A Aziz Q Bennett MI Benoliel R. A classification of chronic pain for ICD-11. Pain. (2015) 156:1003–7. 10.1097/j.pain.0000000000000160 25844555 PMC4450869

[16] Woolf CJ Salter MW. Neuronal plasticity: increasing the gain in pain. Science. (2000) 288:1765–9. 10.1126/science.288.5472.1765 10846153

[17] Ren K Dubner R. Interactions between the immune and nervous systems in pain. Nat Med. (2010) 16:1267–76. 10.1038/nm.2234 20948535 PMC3077564

[18] Guo W Imai S Zou S Yang J.-L. Watanabe M. Altered glial glutamate transporter expression in descending circuitry and the emergence of pain chronicity. Mol Pain. (2019) 15:1–16. 10.1177/1744806918825044 30799685 PMC6348548

[19] De Castro Costa M De Sutter P Gybels J Van Hees J. Adjuvant-induced arthritis in rats: a possible animal model of chronic pain. Pain. (1981). 10:173–85. 10.1016/0304-3959(81)90193-7 7267134

[20] Coderre TJ Wall PD. Ankle joint urate arthritis (AJUA) in rats: an alternative animal model of arthritis to that produced by Freund's adjuvant. Pain. (1987) 28:379–93. 10.1016/0304-3959(87)90072-8 3574965

[21] Zhang RX Ren K. Animal models of inflammatory pain. In: Zhang JM Ma C editors. Animal Models of Pain. New York, NY: Springer Science+Business Media, The Humana Press, Inc. (2010). p. 23–40. 10.1007/978-1-60761-880-5_2

[22] Wang K Luo Y Asaki T Graven-Nielsen T Cairns BE Arendt-Nielsen T. Acid-induced experimental muscle pain and hyperalgesia with single and repeated infusion in human forearm. Scand J Pain. (2017) 17:260–6. 10.1016/j.sjpain.2017.07.012 29229212

[23] Shi C Qiu S Riester SM Das V Zhu B Wallace AA. Animal models for studying the etiology and treatment of low back pain. J Orthop Res. (2018) 36:1305–12. 10.1002/jor.23741 28921656 PMC6287742

[24] Song XJ Hu SJ Greenquist KW Zhang JM LaMotte RH. Mechanical and thermal hyperalgesia and ectopic neuronal discharge after chronic compression of dorsal root ganglia. J Neurophysiol. (1999) 82:3347–58. 10.1152/jn.1999.82.6.3347 10601466

[25] Kim JS Kroin JS Li X An HS Buvanendran A Yan D. The rat intervertebral disk degeneration pain model: relationships between biological and structural alterations and pain. Arthr Res Ther. (2011) 13:R165. 10.1186/ar3485 21996269 PMC3308099

[26] Kawakami M Tamaki T Weinstein JN Hashizume H Nishi H Meller ST. Pathomechanism of pain-related behavior produced by allografts of intervertebral disc in the rat. Spine. (1996) 21:2101–7. 10.1097/00007632-199609150-00009 8893434

[27] Lee M Kim BJ Lim EJ Back SK Lee JH Yu SW. Complete Freund's adjuvant-induced intervertebral discitis as an animal model for discogenic low back pain. Anesth Analg. (2009) 109:1287–96. 10.1213/ane.0b013e3181b31f39 19762759

[28] Xie WR Deng H Li H Bowen TL Strong JA Zhang JM. Robust increase of cutaneous sensitivity, cytokine production and sympathetic sprouting in rats with localized inflammatory irritation of the spinal ganglia. Neuroscience. (2006) 142:809–22. 10.1016/j.neuroscience.2006.06.045 16887276 PMC1661830

[29] Guo W Wang H Zou S Wei F Dubner R Ren K. Long lasting pain hypersensitivity following ligation of the tendon of the masseter muscle in rats: a model of myogenic orofacial pain. Mol Pain. (2010) 6:40. 10.1186/1744-8069-6-40 20633279 PMC2914030

[30] Guo W Wang H Zou S Gu M Watanabe M Wei F. Bone marrow stromal cells produce long-term pain relief in rat models of persistent pain. Stem Cells. (2011) 29:1294–303. 10.1002/stem.667 21630378 PMC3277433

[31] Clauw DJ. Fibromyalgia: a clinical review. JAMA. (2014) 311:1547–55. 10.1001/jama.2014.3266 24737367

[32] Perrot S Cohen M Barke A Korwisi B Rief W Treede D.. The IASP classification of chronic pain for ICD-11: chronic secondary musculoskeletal pain. Pain. (2019) 160:77–82. 10.1097/j.pain.0000000000001389 30586074

[33] Nagakura Y Oe T Aoki T Matsuoka N. Biogenic amine depletion causes chronic muscular pain and tactile allodynia accompanied by depression: a putative animal model of fibromyalgia. Pain. (2009) 146:26–33. 10.1016/j.pain.2009.05.024 19646816

[34] Hernandez-Leon A De la Luz-Cuellar YE Granados-Soto V González-Trujano ME Fernández-Guasti A. Sex differences and estradiol involvement in hyperalgesia and allodynia in an experimental model of fibromyalgia. Horm Behav. (2018) 97:39–46. 10.1016/j.yhbeh.2017.10.011 29080671

[35] Yaffe D Forrest LR Schuldiner S. The ins and outs of vesicular monoamine transporters. J Gen Physiol. (2018) 150:671–82. 10.1085/jgp.201711980 29666153 PMC5940252

[36] Nackley AG Tan KS Fecho K Flood P Diatchenko L Maixner W. Catechol-O-methyltransferase inhibition increases pain sensitivity through activation of both beta2- and beta3-adrenergic receptors. Pain. (2007) 128:199–208. 10.1016/j.pain.2006.09.022 17084978 PMC1905861

[37] Echeverry S Shi XQ Yang M Huang H Wu Y Lorenzo LE. Spinal microglia are required for long-term maintenance of neuropathic pain. Pain. (2017) 158:1792–801. 10.1097/j.pain.0000000000000982 28746078

[38] Lawrence T Gilroy DW Colville-Nash PR Willoughby DA. Possible new role for NF-kappaB in the resolution of inflammation. Nat Med. (2001) 7:1291–7. 10.1038/nm1201-1291 11726968

[39] Okubo M Castro A Guo W Zou S Ren K Wei F. Transition to persistent orofacial pain after nerve injury involves supraspinal serotonin mechanisms. J Neurosci. (2013) 33:5152–61. 10.1523/JNEUROSCI.3390-12.2013 23516281 PMC3640487

[40] Schabrun SM Christensen SW Mrachacz-Kersting N Graven-Nielsen T. Motor cortex reorganization and impaired function in the transition to sustained muscle pain. Cereb Cortex. (2016) 26:1878–90. 10.1093/cercor/bhu319 25609242

[41] Borges do Nascimento IJ Cacic N Abdulazeem HM von Groote TC Jayarajah U Weerasekara I. Novel coronavirus infection (COVID-19) in humans: a scoping review and meta-analysis. J Clin Med. (2020) 9:941. 10.3390/jcm9040941 32235486 PMC7230636

[42] Keeley P Buchanan D Carolan C Pivodic L Tavabie S Noble S. Symptom burden and clinical profile of COVID-19 deaths: a rapid systematic review and evidence summary. BMJ Support Palliat Care. (2020). 10.1136/bmjspcare-2020-002368. [Epub ahead of print]. 32467101

[43] Bonato LL Quinelato V De Felipe Cordeiro PC De Sousa EB Tesch R Casado PL. Association between temporomandibular disorders and pain in other regions of the body. J Oral Rehabil. (2017) 44:9–15. 10.1111/joor.12457 27862166

[44] Almoznino G Zini A Zakuto A Zlutzky H Bekker S Shay B. Cervical muscle tenderness in temporomandibular disorders and its associations with diagnosis, disease-related outcomes, and comorbid pain conditions. J Oral Facial Pain Headache. (2020) 34:67–76. 10.11607/ofph.2374 31465035

[45] Velly AM Look JO Schiffman E Lenton PA Kang W Messner RP. The effect of fibromyalgia and widespread pain on the clinically significant temporomandibular muscle and joint pain disorders–a prospective 18-month cohort study. J Pain. (2010) 11:1155–64. 10.1016/j.jpain.2010.02.009 20466595 PMC2943982

[46] Ayouni I Chebbi R Hela Z Dhidah M. Comorbidity between fibromyalgia and temporomandibular disorders: a systematic review. Oral Surg Oral Med Oral Pathol Oral Radiol. (2019) 128:33–42. 10.1016/j.oooo.2019.02.023 30981530

[47] Giamberardino MA Affaitati G Martelletti P Tana C Negro A Lapenna D. Impact of migraine on fibromyalgia symptoms. J Headache Pain. (2015) 17:28. 10.1186/s10194-016-0619-8 27002510 PMC4803717

[48] Costantini R Affaitati G Wesselmann U Czakanski P Giamberardino MA. Visceral pain as a triggering factor for fibromyalgia symptoms in comorbid patients. Pain. (2017) 158:1925–37. 10.1097/j.pain.0000000000000992 28683025

[49] Vivaldi D Di Giosia M Tchivileva IE Jay GW Slade GD Lim PF. Headache attributed to TMD is associated with the presence of comorbid bodily pain: a case-control study. Headache. (2018) 58:1593–600. 10.1111/head.13404 30178880

[50] Henderson Sr FC Austin C Benzel E Bolognese P Ellenbogen R. Neurological and spinal manifestations of the Ehlers-Danlos syndromes. Am J Med Genet C Semin Med Genet. (2017) 175:195–211. 10.1002/ajmg.c.31549 28220607

[51] Fernández-de-las-Peñas C Galán-del-Río F Fernández-Carnero J Pesquera J Arendt-Nielsen L Svensson P. Bilateral widespread mechanical pain sensitivity in women with myofascial temporomandibular disorder: evidence of impairment in central nociceptive processing. J Pain. (2009) 10:1170–8. 10.1016/j.jpain.2009.04.017 19592309

[52] Clark J Nijs J Yeowell G Goodwin PC. What are the predictors of altered central pain modulation in chronic musculoskeletal pain populations? A systematic review. Pain Phys. (2017) 20:487–500 28934779

[53] Sluka KA Kalra A Moore SA. Unilateral intramuscular injections of acidic saline produce a bilateral, long-lasting hyperalgesia. Muscle Nerve. (2001) 24:37–46. 10.1002/1097-4598(200101)24:1<37::aid-mus4>3.0.co;2-8 11150964

[54] Chai B Guo W Wei F Dubner R Ren K. Trigeminal-rostral ventromedial medulla circuitry is involved in orofacial hyperalgesia contralateral to tissue injury. Mol Pain. (2012) 8:78. 10.1186/1744-8069-8-78 23092240 PMC3484042

[55] Traub RJ Cao DY Karpowicz J Pandya S Ji Y Dorsey SG. A clinically relevant animal model of temporomandibular disorder and irritable bowel syndrome comorbidity. J Pain. (2014) 15:956–66. 10.1016/j.jpain.2014.06.008 24981128 PMC4158438

[56] Ambalavanar R Moutanni A Dessem D. Inflammation of craniofacial muscle induces widespread mechanical allodynia. Neurosci Lett. (2006) 399:249–54. 10.1016/j.neulet.2006.02.003 16510243

[57] Ren K Dubner R. The role of trigeminal interpolaris-caudalis transition zone in persistent orofacial pain. Int Rev Neurobiol. (2011) 97:207–25. 10.1016/B978-0-12-385198-7.00008-4 21708312 PMC3257052

[58] Watkins LR Maier SF. Immune regulation of central nervous system functions: from sickness responses to pathological pain. J Intern Med. (2005) 257:139–55. 10.1111/j.1365-2796.2004.01443.x 15656873

[59] Guo W Wang H Watanabe M Shimizu K Zou S LaGraize SC. Glial-cytokine-neuronal interactions underlying the mechanisms of persistent pain. J Neurosci. (2007) 27:6006–18. 10.1523/JNEUROSCI.0176-07.2007 17537972 PMC2676443

[60] Ross JL Queme LF Cohen ER Green KJ Lu P Shank AT. Muscle IL1β drives ischemic myalgia via ASIC3-mediated sensory neuron sensitization. J Neurosci. (2016) 36:6857–71. 10.1523/JNEUROSCI.4582-15.2016 27358445 PMC4926236

[61] Oliveira-Fusaro MC Gregory NS Kolker SJ Rasmussen L Allen LH Sluka KA. P2X4 receptors on muscle macrophages are required for development of hyperalgesia in an animal model of activity-induced muscle pain. Mol Neurobiol. (2020) 57:1917–29. 10.1007/s12035-019-01852-x 31898158 PMC7124976

[62] Landry RP Jacobs VL Romero-Sandoval EA DeLeo JA. Propentofylline, a CNS glial modulator does not decrease pain in post-herpetic neuralgia patients: in vitro evidence for differential responses in human and rodent microglia and macrophages. Exp Neurol. (2012) 234:340–50. 10.1016/j.expneurol.2011.11.006 22119425

[63] Loggia ML Chonde DB Akeju O Arabasz G Catana C Edwards RR. Evidence for brain glial activation in chronic pain patients. Brain. (2015) 138:604–15. 10.1093/brain/awu377 25582579 PMC4339770

[64] Gupta S Cerosaletti K Long SA. Renegade homeostatic cytokine responses in T1D: drivers of regulatory/effector T cell imbalance. Clin Immunol. (2014) 151:146–54. 10.1016/j.clim.2014.02.007 24576418

[65] Fischer R Sendetski M Del Rivero T Martinez GF Bracchi-Ricard V Swanson KA. TNFR2 promotes Treg-mediated recovery from neuropathic pain across sexes. Proc Natl Acad Sci USA. (2019) 116:17045–50. 10.1073/pnas.1902091116 31391309 PMC6708347

[66] Lees JG Duffy SS Perera CJ Moalem-Taylor G. Depletion of Foxp3+ regulatory T cells increases severity of mechanical allodynia and significantly alters systemic cytokine levels following peripheral nerve injury. Cytokine. (2015) 71:207–14. 10.1016/j.cyto.2014.10.028 25461400

[67] Dong Y Fischer R Naudé PJ Maier O Nyakas C Duffey M. Essential protective role of tumor necrosis factor receptor 2 in neurodegeneration. Proc Natl Acad Sci USA. (2016) 113:12304–9. 10.1073/pnas.1605195113 27791020 PMC5087045

[68] Guo W Imai S Yang JL Zou S Li H Xu H. NF-KappaB pathway is involved in bone marrow stromal cell-produced pain relief. Front Integr Neurosci. (2018) 12:49. 10.3389/fnint.2018.00049 30459569 PMC6232783

[69] Greenspan JD Craft RM LeResche L Arendt-Nielsen L Berkley KJ Fillingim RB. Studying sex and gender differences in pain and analgesia: a consensus report. Pain. (2007) 132(Suppl 1):S26–45. 10.1016/j.pain.2007.10.014 17964077 PMC2823483

[70] Fillingim RB King CD Ribeiro-Dasilva MC Rahim-Williams B Riley JL III. Sex, gender, and pain: a review of recent clinical and experimental findings. J Pain. (2009) 10:447–85. 10.1016/j.jpain.2008.12.001 19411059 PMC2677686

[71] Mogil JS. Sex differences in pain and pain inhibition: multiple explanations of a controversial phenomenon. Nat Rev Neurosci. (2012) 13:859–66. 10.1038/nrn3360 23165262

[72] NIH. Consideration of Sex as a Biological Variable in NIH-Funded Research. Notice Number: NOT-OD-15-102 (2015). Available online at: https://grants.nih.gov/grants/guide/notice-files/not-od-15-102.html (accessed October 6, 2020).

[73] Wijnhoven HA de Vet HC Picavet HS. Explaining sex differences in chronic musculoskeletal pain in a general population. Pain. (2006) 124:158–66. 10.1016/j.pain.2006.04.012 16716517

[74] Yu S Chen C Pan Y Kurz MC Datner E Hendry PL. Genes known to escape X chromosome inactivation predict co-morbid chronic musculoskeletal pain and posttraumatic stress symptom development in women following trauma exposure. Am J Med Genet B Neuropsychiatr Genet. (2019) 180:415–27. 10.1002/ajmg.b.32706 30537437 PMC7138464

[75] Slade GD Ohrbach R Greenspan JD Fillingim RB Bair E Sanders AE. Painful temporomandibular disorder: decade of discovery from OPPERA studies. J Dent Res. (2016) 95:1084–92. 10.1177/0022034516653743 27339423 PMC5004239

[76] Urits I Burshtein A Sharma M Testa L Gold PA Orhurhu V. Low back pain, a comprehensive review: pathophysiology, diagnosis, and treatment. Curr Pain Headache Rep. (2019) 23:23. 10.1007/s11916-019-0757-1 30854609

[77] Häuser W Ablin J Fitzcharles MA Littlejohn G Luciano JV Usui C. Fibromyalgia. Nat Rev Dis Prim. (2015) 1:15022. 10.1038/nrdp.2015.22 27189527

[78] Uhl RL Roberts TT Papaliodis DN Mulligan MT Dubin AH. Management of chronic musculoskeletal pain. J Am Acad Orthop Surg. (2014) 22:101–10. 10.5435/00124635-201402000-00005 24486756

[79] Babatunde OO Jordan JL Van der Windt DA Hill JC Foster NE Protheroe J. Effective treatment options for musculoskeletal pain in primary care: a systematic overview of current evidence. PLoS ONE. (2017) 12:e0178621. 10.1371/journal.pone.0178621 28640822 PMC5480856

[80] Martimbianco ALC Torloni MR Andriolo BN Porfírio GJ Riera R. Neuromuscular electrical stimulation (NMES) for patellofemoral pain syndrome. Cochrane Database Syst Rev. (2017) 12:CD011289. 10.1002/14651858.CD011289.pub2 29231243 PMC6486051

[81] Laimi K Mäkil ä A Bärlund E Katajapuu N Oksanen A Seikkula V. Effectiveness of myofascial release in treatment of chronic musculoskeletal pain: a systematic review. Clin Rehabil. (2018) 32:440–50. 10.1177/0269215517732820 28956477

[82] Pettine KA Suzuki RK Sand TT Murphy MB. Autologous bone marrow concentrate intradiscal injection for the treatment of degenerative disc disease with three-year follow-up. Int Orthop. (2017) 41:2097–103. 10.1007/s00264-017-3560-9 28748380

[83] Wolff M Shillington JM Rathbone C Piasecki SK Barnes B. Injections of concentrated bone marrow aspirate as treatment for discogenic pain: a retrospective analysis. BMC Musculoskelet Disord. (2020) 21:135. 10.1186/s12891-020-3126-7 32111220 PMC7049206

[84] Guo W Imai S Dubner R Ren K. Multipotent stromal cells for arthritic joint pain therapy and beyond. Pain Manag. (2014) 4:153–62. 10.2217/pmt.14.1 24641438

[85] Harrell CR Markovic BS Fellabaum C Arsenijevic A Volarevic V. Mesenchymal stem cell-based therapy of osteoarthritis: current knowledge and future perspectives. Biomed Pharmacother. (2019) 109:2318–26. 10.1016/j.biopha.2018.11.099 30551490

[86] Bastos R Mathias M Andrade R Amaral R.J. F. C. Schott V. Intra-articular injection of culture-expanded mesenchymal stem cells with or without addition of platelet-rich plasma is effective in decreasing pain and symptoms in knee osteoarthritis: a controlled, double-blind clinical trial. Knee Surg Sports Traumatol Arthrosc. (2020) 28:1989–99. 10.1007/s00167-019-05732-8 31587091

[87] Jo CH Chai JW Jeong EC Oh S Yoon KS. Intratendinous injection of mesenchymal stem cells for the treatment of rotator cuff disease: a 2-year follow-up study. Arthroscopy. (2020) 36:971–80. 10.1016/j.arthro.2019.11.120 31805388

[88] Kumar H Ha DH Lee EJ Park JH Shim JH Ahn TK. Safety and tolerability of intradiscal implantation of combined autologous adipose-derived mesenchymal stem cells and hyaluronic acid in patients with chronic discogenic low back pain: 1-year follow-up of a phase I study. Stem Cell Res Ther. (2017) 8:262. 10.1186/s13287-017-0710-3 29141662 PMC5688755

[89] Urits I Capuco A Sharma M Kaye AD Viswanath O Cornett EM. Stem cell therapies for treatment of discogenic low back pain: a comprehensive review. Curr Pain Headache Rep. (2019) 23:65. 10.1007/s11916-019-0804-y 31359164

[90] Guo W Zou S Mohammad Z Wang S Yang J Li H. Voluntary biting behavior as a functional measure of orofacial pain in mice. Physiol Behav. (2019) 204:129–39. 10.1016/j.physbeh.2019.02.024 30797813 PMC6475465

[91] Ren K. Exosomes in perspective: a potential surrogate for stem cell therapy. Odontology. (2019) 107:271–84. 10.1007/s10266-018-0395-9 30324571 PMC6465182

[92] Guo W Chu YX Imai S Yang JL Zou S Mohammad Z. Further observations on the behavioral and neural effects of bone marrow stromal cells in rodent pain models. Mol Pain. (2016) 12:1744806916658043. 10.1177/1744806916658043 27329776 PMC4956005

[93] Guo W Imai S Yang JL Zou S Watanabe M Chu YX. *In vivo* immune interactions of multipotent stromal cells underlie their long-lasting pain-relieving effect. Sci Rep. (2017) 7:10107. 10.1038/s41598-017-10251-y 28860501 PMC5579160

[94] Harting MT Jimenez F Xue H Fischer UM Baumgartner J Dash PK. Intravenous mesenchymal stem cell therapy for traumatic brain injury. J Neurosurg. (2009) 110:1189–197. 10.3171/2008.9.JNS08158 19301973 PMC2889620

[95] Lee RH Pulin AA Seo MJ Kota DJ Ylostalo J Larson BL. Intravenous hMSCs improve myocardial infarction in mice because cells embolized in lung are activated to secrete the anti-inflammatory protein TSG-6. Cell Stem Cell. (2009) 5:54–63. 10.1016/j.stem.2009.05.003 19570514 PMC4154377

[96] Jung JW Kwon M Choi JC Shin JW Park IW Choi BW. Familial occurrence of pulmonary embolism after intravenous, adipose tissue-derived stem cell therapy. Yonsei Med J. (2013) 54:1293–6. 10.3349/ymj.2013.54.5.1293 23918585 PMC3743204

[97] Chen G Park CK Xie RG Ji RR. Intrathecal bone marrow stromal cells inhibit neuropathic pain via TGF-β secretion. J Clin Invest. (2015) 125:3226–40. 10.1172/JCI80883 26168219 PMC4563753

[98] Henriksson HB Papadimitriou N Hingert D Baranto A Lindahl A Brisby H. The traceability of mesenchymal stromal cells after injection into degenerated discs in patients with low back pain. Stem Cells Dev. (2019) 28:1203–11. 10.1089/scd.2019.0074 31237488

[99] Waterman RS Tomchuck SL Henkle SL Betancourt AM. A new mesenchymal stem cell (MSC) paradigm: polarization into a pro-inflammatory MSC1 or an Immunosuppressive MSC2 phenotype. PLoS ONE. (2010) 5:e10088. 10.1371/journal.pone.0010088 20436665 PMC2859930

[100] Zhu C Wang K Chen Z Han Y Chen H Li Q. Antinociceptive effect of intrathecal injection of miR-9-5p modified mouse bone marrow mesenchymal stem cells on a mouse model of bone cancer pain. J Neuroinflammation. (2020) 17:85. 10.1186/s12974-020-01765-w 32178691 PMC7075036

